# Serum Metabolic Correlates of the Antibody Response in Subjects Receiving the Inactivated COVID-19 Vaccine

**DOI:** 10.3390/vaccines10111890

**Published:** 2022-11-09

**Authors:** Yi Zhang, Qiaoyan Yue, Haojing Zhu, Jieyu Song, Dingding Li, Wen Liu, Shujun Jiang, Ning Jiang, Chao Qiu, Jingwen Ai, Yanliang Zhang, Wenhong Zhang

**Affiliations:** 1 Department of Infectious Diseases, Shanghai Key Laboratory of Infectious Diseases and Biosafety Emergency Response, Huashan Hospital, Fudan University, Shanghai 200040, China; 2 Nanjing Hospital of Chinese Medicine Affiliated to Nanjing University of Chinese Medicine, Nanjing 210006, China; 3 Nanjing Research Center for Infectious Diseases of Integrated Traditional Chinese and Western Medicine, Nanjing 210006, China; 4 Shanghai Huashen Institute of Microbes and Infections, No. 6 Lane 1220 Huashan Rd., Shanghai 200031, China

**Keywords:** COVID-19, vaccine, humoral immune response, serum metabolism, regulation

## Abstract

Background: Metabolites are involved in biological process that govern the immune response to infection and vaccination. Knowledge of how metabolites interact with the immune system during immunization with the COVID-19 vaccine is limited. Here, we report that the serum metabolites are correlated with the magnitude of the antibody response in recipients receiving the inactivated COVID-19 vaccine, which provides critical information for studying metabolism regarding the human immune response to vaccination. Methods: 106 healthy volunteers without history of SARS-CoV-2 infection or vaccination were prospectively enrolled to receive the primary series of two doses of inactivated whole-virion SARS-CoV-2 vaccine. The serum samples were collected 2–4 weeks after the second dose. The magnitude of the anti-RBD antibody was quantified using surrogate virus neutralization tests. The profile of metabolites in serum was identified using untargeted metabolomics analysis. Results: The level of anti-RBD antibody 14–28 days after the second dose was significantly elevated and its interpersonal variability was diverse in a wide range. Thirty-two samples at extremes of the anti-RBD antibody titer were selected to discover the metabolic correlates. Two hundred and fifteen differential metabolites associated with antibody response independent of body mass index were identified. Pregnenolone and sphingolipid metabolism might be involved in the modulation of the human antibody response to the inactivated COVID-19 vaccine. Conclusion: We discovered key metabolites as well as those with a related functional significance that might modulate the human immune response to vaccination.

## 1. Introduction

The adaptive immune response is highly dynamic. Upon exposure to antigens, T and B cells undergo activation, extensive clonal expansion in the primary effector phase, and the generation of memory cells that persist long-term to fight against invading pathogens carrying previously encountered antigens [1,2]. This process is tightly orchestrated by antigen amount, costimulatory signal, and nutrient availability in the microenvironment. An improper profile of essential metabolites might impair the adaptive immune response, while the helpful additive metabolites may promote not only the magnitude of the immune response but also the longevity of the immune memory. With the feasibility of the research tools and experimental system, it is well known that the cellular metabolism is a critical mechanism to alter immune cell activation, differentiation, and function. However, only a few studies have worked on immunometabolism at an organismal level are emerging, and most of them are focused on the perturbation of metabolites in pathological damage. Taking COVID-19 or pulmonary tuberculosis [3,4] as an example, some metabolites are tightly correlated with immune response as a result of the product of an inflammatory signal, in which metabolism is very useful for discovering disease-related biomarkers. In these circumstances of severe disease, metabolic cues are overly disturbed by pathological inflammatory responses, cell necrosis, and tissue distortion, which is the consequences of disease pathology. Therefore, it is useful to better understand the pathophysiology of the disease, identify new biomarkers, and elucidate targets for host-directed therapeutics [2]. In contrast, in the context of vaccination, the immune response is almost given by the administered vaccine, which provides an important opportunity to unveil the role of organismal metabolism in shaping immune function.

There was a rollout of vaccines to curb the spread of the COVID-19 pandemic. This allowed us an opportunity to discover the serum metabolic correlations of the antibody response to the COVID-19 vaccine. There are several advantages of this: first, preexisting antibodies in people are rare, which guarantees the accurate and reliable measurement of the readout of the experimental end point, in lieu of a fold increase in the evaluation of the flu vaccine. Second, the tight public health measures employed in China warrant that the immune response to COVID-19 is due to vaccination, not natural infection. Third, there are a broad range of interindividual variations in adult recipients, which is good for studying the serum metabolic correlations of the antibody response to the COVID-19 vaccine. 

Coronavirus disease 2019 (COVID-19), caused by SARS-CoV-2, led to more than 0.58 billion COVID-19 infections and 6.4 million deaths by 8 August 2022 [5]. The humoral responses are influenced by several factors, including age, sex, and underlying conditions. Spike-specific IgG antibodies in the serum are lower in COVID-19 patients with a high body mass index (BMI) [6]. The antibodies in COVID-19 obese patients were reported to be negatively associated with metabolic and pro-inflammatory markers [7]. 

A number of studies have shown that the metabolic environment plays an important role in the function of immune cells, for example, influencing the activation, proliferation, and differentiation of immune cells by changing the intakes of the metabolites and the signal transduction pathway. Metabolism in COVID-19 has received plenty of attention as it plays an important role in the development of many diseases. Whole-spectrum analysis of metabolites in different severity levels of COVID-19 patients found that with the aggravation of COVID-19, the types of metabolites changing in the plasma gradually increased [8]. A study in South Korea found a significant change in the plasma metabolic components in COVID-19 patients, which was closely associated with disease severity [9]. The Dengue virus vaccine elicited an effector/memory-associated transcriptional signature that was reported to be dominated by a metabolic transcription program [10]. Nevertheless, Bacille Calmette-Gue’ rin (BCG) vaccine could induce metabolic changes through an elevated concentration of lysophospholipids in the plasma [11]. 

Although the metabolites in COVID-19 patients and other diseases have been studied, the effects of metabolism on immunity were mostly focused on cellular immunity. The relationship of metabolites related to B-cell-mediated humoral immunity of COVID-19 vaccination has been studied far less. In this paper, we provide an understanding of the regulatory role of the serum metabolism in the humoral immune response of vaccines based on an analysis of the serum metabolism in healthy adults after two doses of the COVID-19 vaccination.

## 2. Methods

### 2.1. Sample Collection 

In this prospective cohort study, healthy volunteers were enrolled from the Nanjing Hospital of Chinese Medicine Affiliated to Nanjing University of Chinese Medicine. All participants received two doses of inactivated whole-virion SARS-CoV-2 vaccines (CoronaVac or BBIBP-CorV) two to four weeks before enrollment. The second-dose vaccination date was from 1 August to 11 October 2021. We collected information on the basic characteristics (age, sex, body mass index (BMI), and body fat rate) and comorbidities (hypertension, diabetes, cardiovascular disease, chronic kidney diseases, and other diseases). The study protocol and informed consent form were approved by the Ethics Committees of the Nanjing Hospital of Chinese Medicine (2021162).

### 2.2. Detection of Anti-SARS-CoV-2 Receptor Binding Domain (RBD) Neutralizing Responses, Antibodies, and IgG

The serum samples were isolated from centrifuged blood samples. We assessed the anti-RBD responses using a plasma surrogate virus neutralization test, as well as IgM and IgG tests. The neutralizing titer was measured using the SARS-CoV-2 Neutralizing Ab detection kit (PerkinElmer SuperFlex Anti-SARS-CoV-2 Neutralizing Ab Kit, SDX-57042). The anti-RBD total antibody and IgG was performed using the PerkinElmer SuperFlex Anti-SARS-CoV-2 total antibody and IgG Kit.

### 2.3. Liquid Chromatography–Mass Spectrometry (LC-MS) Sample Procedure 

Among the participants, 14–28 days after vaccination, we selected seven subjects from the top 30% neutralizing antibody titer and top 30% BMI (H-H group), nine subjects from the bottom 30% neutralizing antibody titer and bottom 30% BMI (L-L group), eight subjects with the top 30% neutralizing antibody titer and bottom 30% BMI (NH-BL group), and nine subjects with the bottom 30% neutralizing antibody titer and top 30% BMI (NL-BH group). The H-H group and NH-BL group belonged to the high-antibody group, and the L-L and NL-BH group belonged to the low-antibody group. Similarly, the 32 samples could be classified into high-BMI and low-BMI groups. 

For the preparation of samples, an aliquot of 100 μL of the sample serum (balanced to room temperature) was added with 20 μL L-2-chlorophenylalanine (Shanghai HC Biotech Co., Ltd., Shanghai, China, 0.06 mg/mL) eddy shock for 10 s. Then, 300 μL of protein precipitator methanol-acetonitrile (*v*/*v* = 2/1) was added to the mixture and oscillated for 1 min. Ultrasonic extraction occurred with an ice water bath for 10 min, and it was left to stand at −20 °C for 30 min. Then, the mixture was centrifuged for 10 min (13,000 RPM, 4 °C), and 200 μL supernatant was put into the LC-MS vial and dried. It was then dissolved with 300 μL methanol–water (*v*/*v* = 1/4). After standing at −20 °C for 2 h, it was centrifuged for 10 min (13,000 RPM, 4 °C). Finally, 150 μL of the supernatant was absorbed with a syringe, filtered using a 0.22 μm organic phase pinhole filter, and transferred to an LC-MS vial for subsequent analysis. Quality control (QC) occurred through using an equal volume of mixture of extracts from all of the samples.

The ACQUITY UPLC I-Class PLUS (Waters) was equipped with a Q Exactive mass spectrometer (Thermo Scientific) attached to a heated electrospray ionization (ESI) source to analyze the ESI positive and ESI negative ion mode of the metabolic spectrum. An ACQUITY UPLC HSS T3 column was used in the positive and negative modes.

### 2.4. Data Processing and Statistical Analysis 

The acquired LC-MS raw data were identified by progenesis QI (Waters Corporation, Milford, NH, USA) Data Processing Software, based on public databases such as http://www.hmdb.ca/ (accessed on 18 December 2018) and http://www.lipidmaps.org/ (accessed on 2 October 2019) and through self-built databases. The metabolic compound identifications were based on the Human Metabolome Database (HMDB), LIpidmaps (V2.3), Metlin, EMDB, PMDB, and self-built databases. Principle component analysis (PCA) and orthogonal partial least-squares-discriminant analysis (OPLS-DA) were carried out to visualize the metabolic alterations among the experimental groups, after mean centering (Ctr) and Pareto variance (Par) scaling, respectively. The heatmaps and correlations among the metabolites were generated using R package (ggplot2). Different expression analyses were performed on the metabolites between the antibody groups and BMI groups. The correlation between the neutralizing antibody, BMI, and metabolites was calculated using a mixed linear regression model, as follows:


(1) y=α BMI+β antibody+γ BMI∗antibody+ϵ

Additionally, the interaction index and *p* value were obtained. For the clinical characteristics, continuous variables were presented through mean values and standardized deviation or median (interquartile range, IQR) when appropriate. Counts (percentage) were used in categorial variables. Comparisons between two groups were performed by Student’s test, Mann–Whitney U test as appropriate. RandomForest was used to calculate the out-of-bag error for predicting high- and low-antibody groups using the top50 differential metabolites. The statistical analyses were performed in Stata (version 14.1) and figures were generated using Prism (version 8) and Rstudio (version 1.2).

## 3. Results 

### 3.1. Demographic Characteristics and Immune Response after Two-dose Vaccination

**Figure 1:**
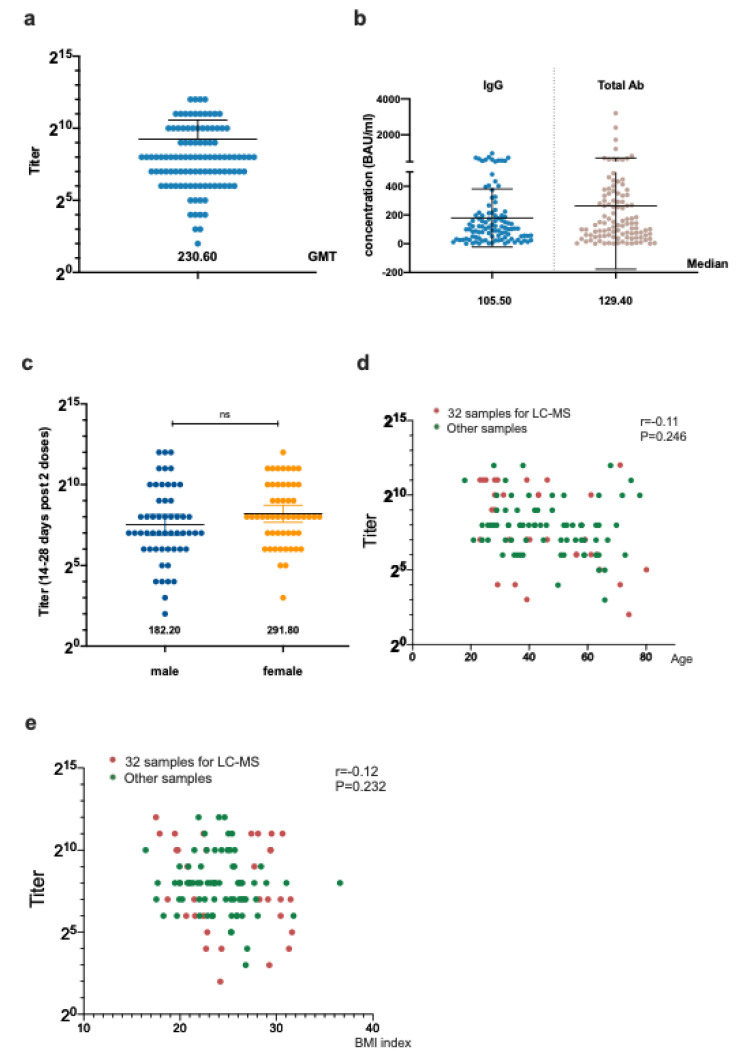
The humoral response of the enrolled patients and the influence of different factors. (**a**) The anti−RBD neutralizing titer in all enrolled participants. (**b**) The anti−RBD total antibody and IgG concentration in all enrolled participants. (**c**) The anti−RBD neutralizing titer in 106 participants collected 14−28 days post vaccination according to sex. (**d**) The correlation of the anti−RBD neutralizing titer and age. (**e**) The correlation of the anti−RBD neutralizing titer and BMI.

The metabolic phenotypes of the Zostavax vaccine recipients on day 7 were recovered, similar with those on day 0 following vaccination [12]. A total of 106 samples from health care providers were collected post vaccination from patients who were enrolled from August 2021 to October 2021, including 53 (50.00%) male patients who were on average 45.8 ± 15.7 years old. The average BMI was 24.6 ± 4.8. The sVNT geometric mean neutralizing titer (GMT) was 230.60. The anti-RBD IgG and total antibody level 14–28 days post two-dose vaccination in these participants was 105.50 [IQR 37.16–213.7] BAU/mL and 129.40 [IQR 50.40–333.70] BAU/mL (Figure 1 and Figure S1). The IgG and total antibody levels were similar in the male and female groups. Despite a significant *p* value, we observed the trend that a BMI < 24 might have a higher IgG and total antibody. Additionally, lower IgG and antibodies were found in the older participants (Supplementary Figure S1).

### 3.2. Metabolites Detection among Two-dose Vaccines 

**Figure 2:**
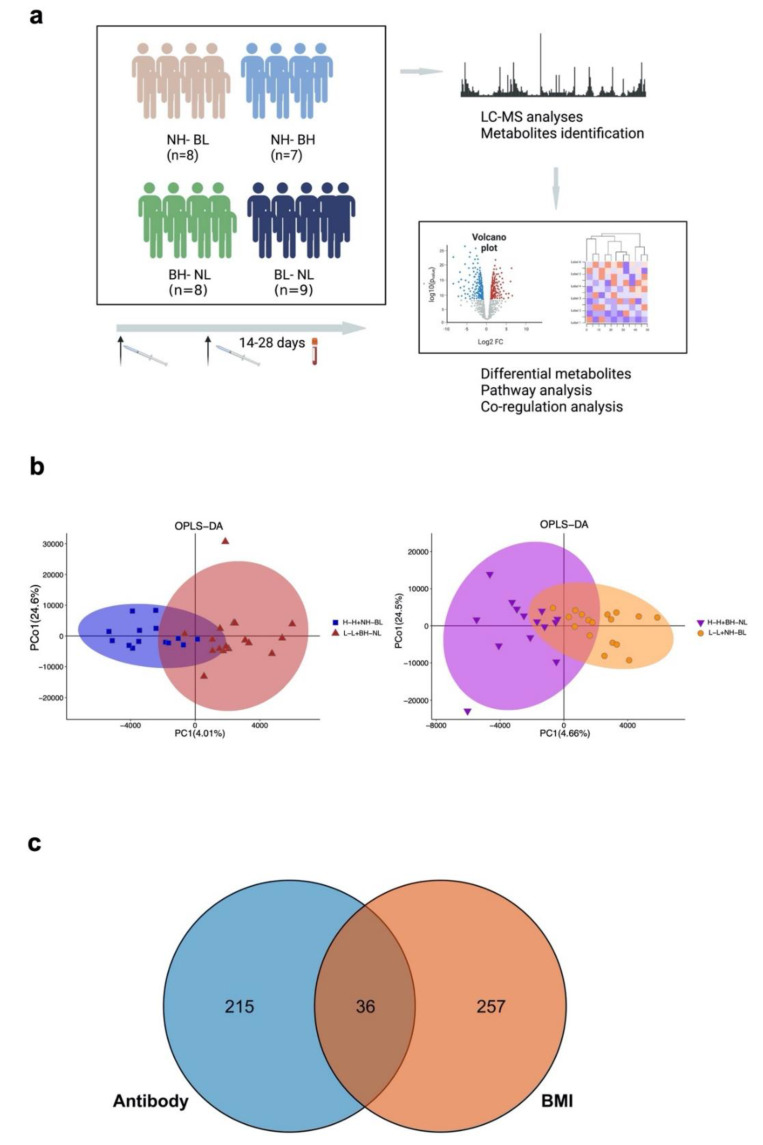
LC–MS study design and metabolite distribution. (**a**) The study design and flowchart of the LC–MS analyses. (**b**) The PCA analyses according to the antibody level (H–H/NH–BL group vs. L–L/BH–NL group) and BMI (H–H/BH–NL group vs. L–L/NH–BL group). (**c**) The OPLS–DA analyses according to the antibody level (H–H/NH–BL group vs. L–L/BH–NL group) and BMI (H–H/BH–NL group vs. L–L/NH–BL group). The overlapped parts were metabolites with *p* values in both the antibody groups and BMI groups.

In the samples processed for LC-MS, 5854 metabolites were identified in 17,072 peaks measured using the LC-MS platforms in 32 serum samples of the cohort (Figure 2a). We then analyzed them according to two factors, namely the antibody level and BMI. From the PCA analyses (Supplementary Figure S2), it was found that the high and low antibody groups and the high and low BMI groups had a similar distribution of metabolites. Using OPLS-DA, we found distinct patterns of metabolism according to the antibodies and BMI, with PCo1 of 24.6% and 24.5%, respectively (Figure 2b). 

For the differential metabolites, according to the antibody level and BMI, 251 metabolites were found to be expressed significantly differently in high and low antibody groups, and 293 metabolites were found to be expressed significantly differently in the high and low BMI groups. Of these metabolites, 36 were found in both of the comparisons (Figure 2c). 

### 3.3. Differential Expression Metabolites between High- and Low-Antibody Groups

**Figure 3:**
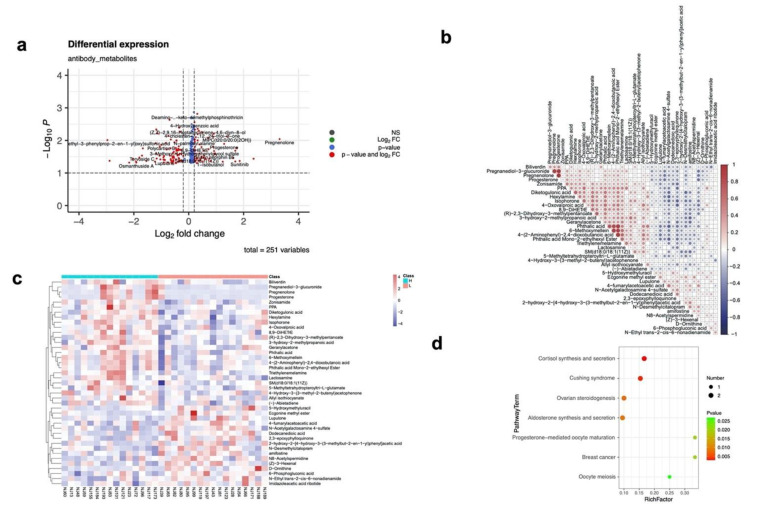
The differential metabolites in the high− and low−antibody groups. (**a**) The differential metabolite expression identified in the high- and low-antibody groups. The x axis shows the log2 fold change in metabolites for two groups (high antibody group vs. low antibody group), and the y axis represents the −log10 *p* values of the metabolites. (**b**) The similarity of the differential metabolite distribution identified in the high− and low−antibody groups. (**c**) The heatmap of differential metabolites distribution in the high− and low−antibody groups. (**d**) The KEGG enrichment pathways of the differential metabolite distribution identified in the high− and low−antibody groups.

A total of 251 metabolites were found to be differently expressed in the high and low antibody groups, including 168 upregulated and 83 downregulated metabolites in the high antibody group (Figure 3a). Among the 215 metabolites that only differed in the high- and low-antibody groups (Supplementary dataset 1), 41 metabolites could be found in the KEGG database. By using the top 50 out of 215 metabolites, the out-of-bag error of the high- and low-antibody group was 40% and 17.6%, respectively. 

The similarity of these metabolites (Figure 3b) showed that pregnanediol-3-glucuronide and pregnenolone shared a high similarity (0.88), and that phthalic acid, 6-methoxymellein, and phthalic acid mono-2-ethylhexyl ester were similar as well (>0.75). The pathway analyses identified seven pathways significantly enriched in the high antibody group, all originating from the metabolite pregnenolone (Figure 3c,d). The fold change of Pregnenolone reached 9.92 with *p* = 0.009. Pregnenolone was obviously higher in the high antibody group than in the low group for females.

For the top 20 upregulated enriched pathways (Supplementary Figures S3 and S4), the pathway Necroptosis/Sphingolipid signaling pathway/Sphingolipid metabolism were related to the metabolite SM(d18:0/18:1(11Z)), which was reported to modulate the immune response. The drug metabolism (cytochrome P450) pathway was found to be downregulated in the high-antibody group. 

### 3.4. Differential Expression Metabolites between the High- and Low-BMI Groups

**Figure 4:**
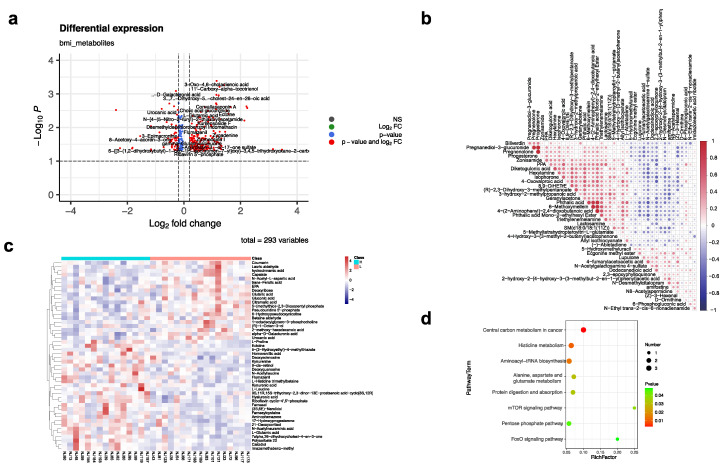
The differential metabolites in the high− and low−BMI groups. (**a**) The differential metabolites expression identified in the high− and low−BMI groups. The x axis shows the log2 fold change of the metabolites in two groups (high antibody group vs. low antibody group), and the y axis represents the −log10 *p* values of the metabolites. (**b**) The similarity of the differential metabolite distribution identified in the high− and low−BMI groups. (**c**) The heatmap of differential metabolites distribution in the high− and low−BMI groups. (**d**) The KEGG enrichment pathways of the differential metabolite distribution identified in the high− and low−BMI groups.

We identified 293 metabolites expressed differently in the high and low antibody groups, including 165 upregulated and 128 downregulated metabolites in the high antibody group (Figure 4a). Among the 257 metabolites that only differed in the high-and low-BMI groups (Supplementary dataset 2), 48 metabolites could be found in the KEGG database. The similarity of these metabolites (Figure 4b) showed that hydrocinnamic acid and Capsiate shared a high similarity (0.89), and Calcidiol, Polysorbate, and mazamethabenz-methyl had a high correlation as well. 

Compared to the low-BMI group, several pathways were upregulated in the high-BMI group, including central carbon metabolism; aminoacly-tRNA biosybthesis; Alanine, asparate, and glutamate metabolism; protein digestion and absorption; mTOR signaling pathway; and the FoxO signaling pathway. These pathways focused on the metabolites L-Leucine, L-Proline, and L-Glutamic acid (Figure 4c,d). The pentose phosphate pathway (Deoxyribose) and Histine metabolism pathway (L-Glutamic acid, L-Histidine trimethylbetaine, and Urocanic acid) were downregulated in the high BMI group. 

### 3.5. Interaction between the Antibodies and BMI Mediated by Metabolites

Through mixed linear regression model analyses, we further identified the interaction regulated role of metabolism played in antibody groups and BMI groups (Table 1). Among the overlapped 36 metabolites, 8 were found to be interacted (interaction *p* value < 0.05) between the antibody groups and BMI groups, presenting obvious differences in both groups. A total of six out of eight metabolites (2-Octen-4-one; 1-Phenyl-1-pentanone; Tetraethylene glycol; 9,12-dioxo-dodecanoic acid; 3-Ethyltridecan-2-one; Nitrosobenzene) were upregulated in the high-antibody group and low-BMI group simultaneously. Cholic acid glucuronide was downregulated in the high-antibody and low-BMI group. 

## 4. Discussion

Here, we performed a prospective study to learn about the humoral immune responses after two doses of COVID-19 inactivated vaccines, and selected 32 serum samples for LC-MS to learn about the metabolic regulatory role in antibody response. Overall, our study discovered key metabolites as well as related biological functions concerning the COVID-19 vaccination and host response, which helped the potential biomarkers for predicting the immune response and for understanding the underlying mechanism. 

Age and sex are important natural factors that influence vaccine responses. Similar to the previous reports [13,14], we found a weakened humoral immune response to vaccination related to aging in this study. Additionally, our study found that female participants tended to have relatively higher neutralizing antibodies, although without a significant *p* value. This trend was consistent with several reports regarding to COVID-19 vaccination [13,14]. The vaccination season in many vaccines is correlated with the antibody responses, such as for the pertussis, ppv23, rabies, and salmonella typhi vaccines [15,16]. Nevertheless, in some other vaccines such as those for diphtheria, Hepatitis B, and tetanus, there is no association between the month of administration and antibody responses [16]. Studies have revealed that those vaccinated in the winter are more likely to experience a higher pathogen interference, which might be associated with microbes and metabolites [17]. However, the mechanism of response for differences according the month of administration remains unknown. The participants of this study were vaccinated with a booster in the period of August 2021 to October 2021, with no considerable differences found.

Apart from being precursors of some steroid hormones, pregnenolon promotes the degradation of key proteins in innate immune signaling to suppress inflammation, inhibiting the secretion of tumor necrosis factor-α and interleukin-6 through TLR2 and TLR4 signaling pathways [18,19]. Progesterone has been reported to regulate neural development and T-cell differentiation, regardless of gender [20,21]. In addition to its role in maintaining pregnancy in females, progesterone is also produced and functions in males [20]. Previous studies have confirmed that progesterone could induce downstream antiviral genes and promotes an innate antiviral response against SARS-CoV-2 infection [22]. SM(d18:0/18:1(11Z)) is located at the Sphingolipid signaling pathway, and regulates T cell apoptosis, Th1 versus Th2 T cell differentiation, and phagocytosis [23]. Recent studies have highlighted the crucial role of sphingolipids in the innate immunity against infecting pathogens as well [24]. Additionally, sphingolipid plays an important role in host–microbe interactions. Gut microbes could produce SM(d18:0/18:1(11Z)), for which the bacteroide-derived sphingolipids are critical for maintaining intestinal homeostasis and symbiosis [25,26].

Several studies of the hepatitis vaccine have shown that an increased BMI is negatively correlated with antibody titer levels in the humoral immune response to the vaccine [27,28,29]. However, subjects with a higher BMI had higher antibody titers in the early stage of vaccination in the trivalent inactivated influenza vaccine (TIV) study, but a great decline in the influenza antibody titers [30]. The immune protection established by the active immunization of vaccination mainly comes from two aspects: one is humoral immune protection produced by antibodies; the other is cellular immune protection mediated by CD8 positive cells. Compared with being a healthy weight, obesity (BMI of 30 or higher) after vaccination with the influenza vaccine produced less specific CD8 T cells and the function of secreting IFN-γ was reduced [30]. The mechanism of immune responses in different BMI groups remains unclear. One perspective is that increased leptin levels accompanying leptin resistance in obesity cause harmful effects on immune signal transduction [31]. However, a decrease in the serum leptin level is related to a decreased immune response for healthy people to influenza or hepatitis B virus vaccine because leptin promotes Tfh cell differentiation and the secretion of IL-21 via the STAT3 and mTOR pathways, thus regulating Tfh cell’s function for supporting antibody affinity maturation and memory formation. A low leptin is related to malnutrition and an increased risk of infection. Therefore, in the perspective of humoral immunity and cellular immune response, a BMI of 30 or higher is detrimental to the immune response to vaccination, as well as for a BMI under 18.5.

Cytochrome P450 was found to be lower in the low antibody group compared with the high antibody group. The Cytochrome-P-450 enzymes (CYP) are among the most important xenobiotic-metabolizing enzymes, which produce reactive oxygen species (ROS) as a result of metabolizing xenobiotics [32]. CYP450 is involved in apoptosis, the activation of antigen presenting cells, and the initiation or amplification of diverse immunologic reactions [33].

The coregulatory functions of several metabolites were found in the antibody responses and BMI. Serum metabolomics in some diseases that may be caused by inflammatory factors, such as inflammatory bowel disease and glaucoma, show that the proinflammatory role of cholic acid glucuronide is associated with an inflammatory response [34,35]. Therefore, in the high BMI group, there may be an inflammatory state induced by this kind of substance that reduces the specific immune response to the vaccine. Fatty acids and conjugates influence the effector and regulatory functions of innate and adaptive immune responses, and play important roles in the modulation of immune responses in healthy and diseased cells [36]. Nevertheless, oxygenated hydrocarbons were observed to be downregulated in the high antibody level group, which has been reported to induce IL-1β, IL-6, and TNF-α production. This mechanism might come from interfering with TCDD, leading to the expression of CYP1A1 and CYP450, the metabolite we identified as being downregulated in the high antibody group above [37]. 3-Oxo-5β-chol-1-en-24-oic acid is down regulated in the high-antibody group and up regulated in the low-BMI group, and might belong to bile acid. The function of this metabolite remained unclear, and could be investigated in the future.

This study has several limitations. First, the limited sample size might not show the whole spectrum of metabolism profiling, and large-scale multi-center analyses could be performed in the future. Second, our enrolled groups had not done sex matching. Third, the cytokines were not tested in our analysis, and we would carry out this testing in the future to provide more immune response data. The mechanism of CYP450 and oxygenated hydrocarbons should be validated later. 

In conclusion, we discovered key metabolites as well as related biological functions concerning the COVID-19 vaccine induced host response. Distinct metabolic profiles highlighted that the elevated pregnenolone metabolism and sphingolipids metabolism, and decreased CYP450 metabolism, could as predictive markers of a COVID-19 vaccine humoral immune response.

## Figures and Tables

**Table 1:** The interaction between antibodies and BMI mediated by metabolites.

Name	Subclass	Antibody *p* Value	High-Antibody Group	BMI *p* Value	Low BMI Group	Interaction *p* Value
2-Octen-4-one	Carbonyl compounds	0.001	up	0.047	up	0.041
1-Phenyl-1-pentanone	Carbonyl compounds	0.008	up	0.024	up	0.029
Tetraethylene glycol	Ethers	0.026	up	0.023	up	0.020
9,12-dioxo-dodecanoic acid	Fatty Acids and Conjugates	0.042	up	0.011	up	0.003
3-Ethyltridecan-2-one	Oxygenated hydrocarbons	0.033	up	0.001	up	0.012
Cholic acid glucuronide	Steroidal glycosides	0.023	down	0.002	down	0.048
3-Oxo-5β-chol-1-en-24-oic Acid	Unclassified	0.018	down	0.003	up	0.024
Nitrosobenzene	Unclassified	0.015	up	0.018	up	0.045

## Data Availability

Available through corresponding author emails.

## Supplementary Materials

The following supporting information can be downloaded at: https://www.mdpi.com/article/10.3390/vaccines10111890/s1, Figure S1: Subgroup analyses of Nab, IgG and total antibody; Figure S2: The PCA analyses according to antibody level (H-H/NH-BL group vs. L-L/BH-NL group) and BMI index (H-H/ BH-NL group vs. L-L/ NH-BL group); Figure S3: The upregulated top 20 KEGG pathways of differential metabolites in antibody high group compared to antibody low group; Figure S4: The downregulated top 20 KEGG pathways of differential metabolites in antibody high group compared to antibody low group. Supplementary dataset 1 and dateset 2 are available through corresponding author.
