# Supplementary material for: Serum Metabolic Correlates of the Antibody Response in Subjects Receiving the Inactivated COVID-19 Vaccine
Source: Vaccines (Basel). 2022 Nov 9;10(11):1890. doi: 10.3390/vaccines10111890 (PMC9699138; doi:10.3390/vaccines10111890)
Supplement: Supplementary file 1 [file vaccines-10-01890-s001.zip › Supplementary materials_revised.pdf]

Supplementary materials

Supplementary Figure S1. Subgroup analyses of Nab, IgG and total antibody.

Supplementary Figure S2. The PCA analyses according to antibody level (H-H/NH-BL group vs. L-L/BH-NL group) and BMI index (H-H/ BH-NL group vs. L-L/ NH-BL group)

Supplementary Figure S3. The upregulated top 20 KEGG pathways of differential metabolites in antibody high group compared to antibody low group.

Supplementary Figure S4. The downregulated top 20 KEGG pathways of differential metabolites in antibody high group compared to antibody low group.

Supplementary Figure S1.

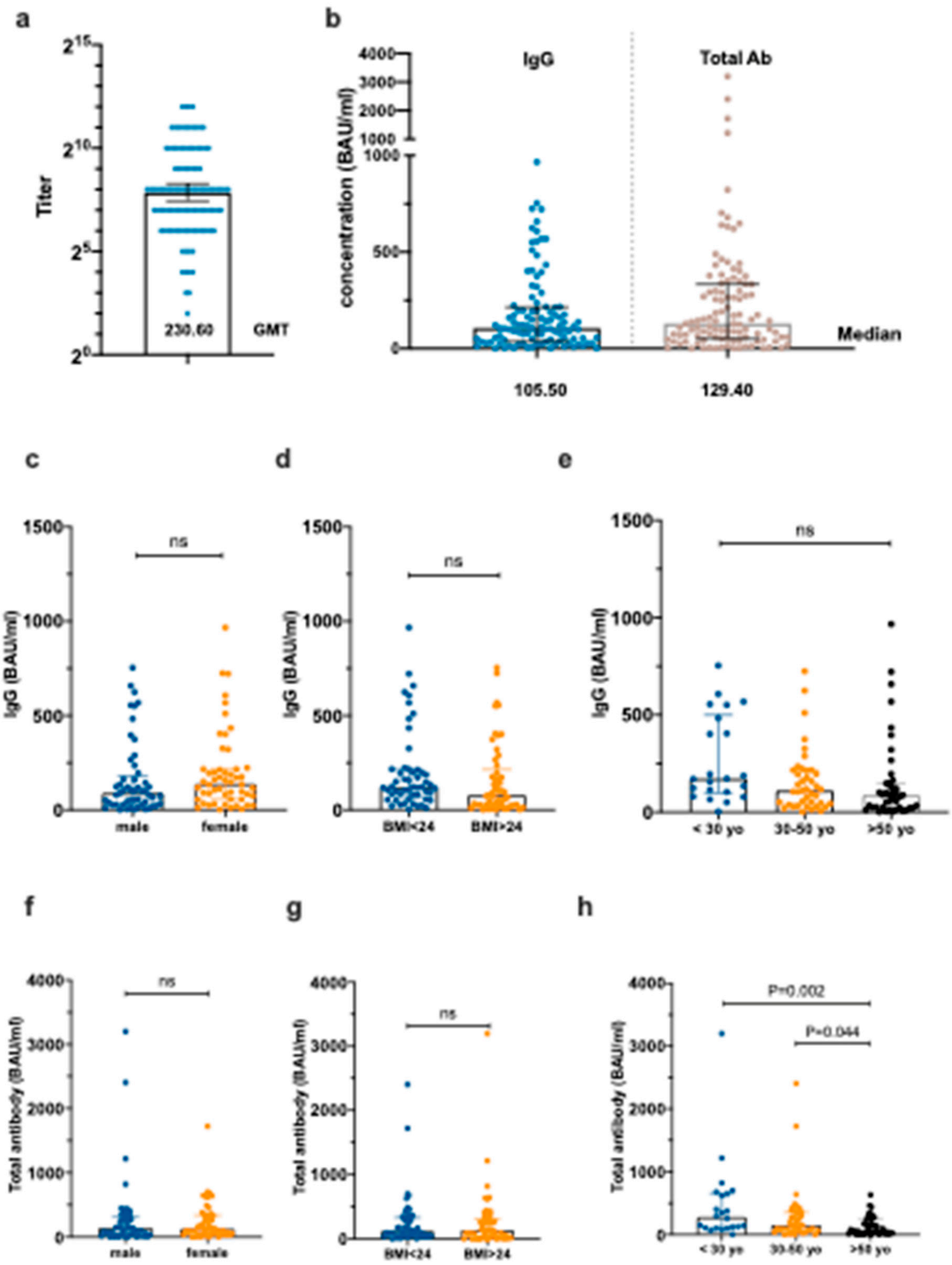

Supplementary Figure S2. The PCA analyses according to antibody level (H-H/NH-BL group vs. L-L/BH-NL group) and BMI index (H-H/ BH-NL group vs. L-L/ NH-BL group)

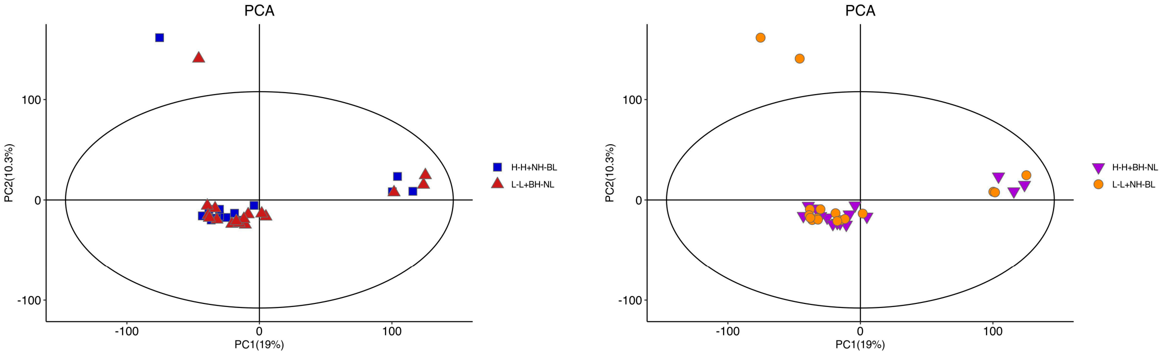

Supplementary Figure S3. The upregulated top 20 KEGG pathways of differential metabolites in antibody high group compared to antibody low group.

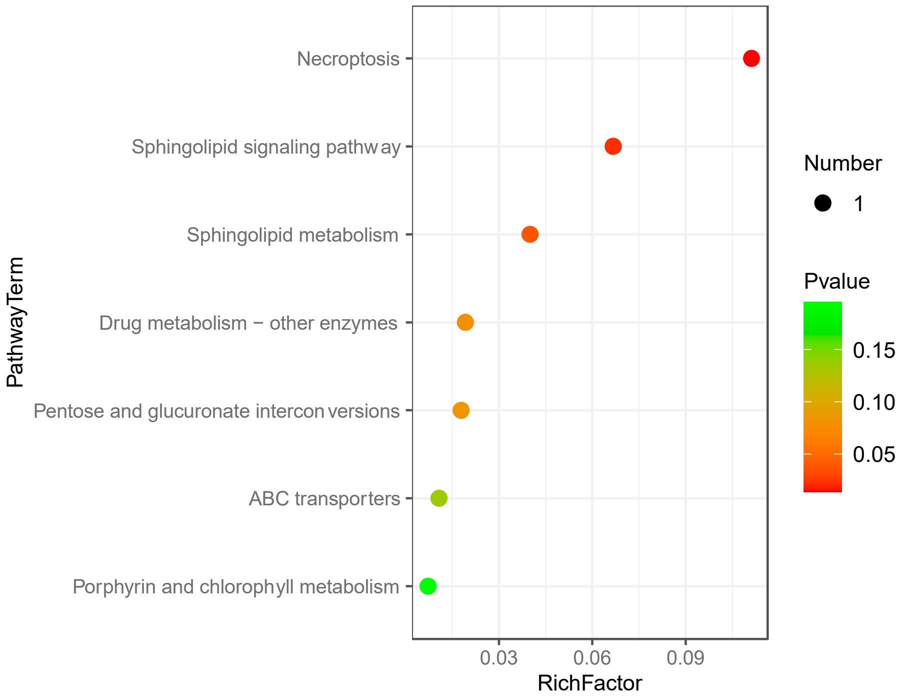

Supplementary Figure S4. The downregulated top 20 KEGG pathways of differential metabolites in antibody high group compared to antibody low group.

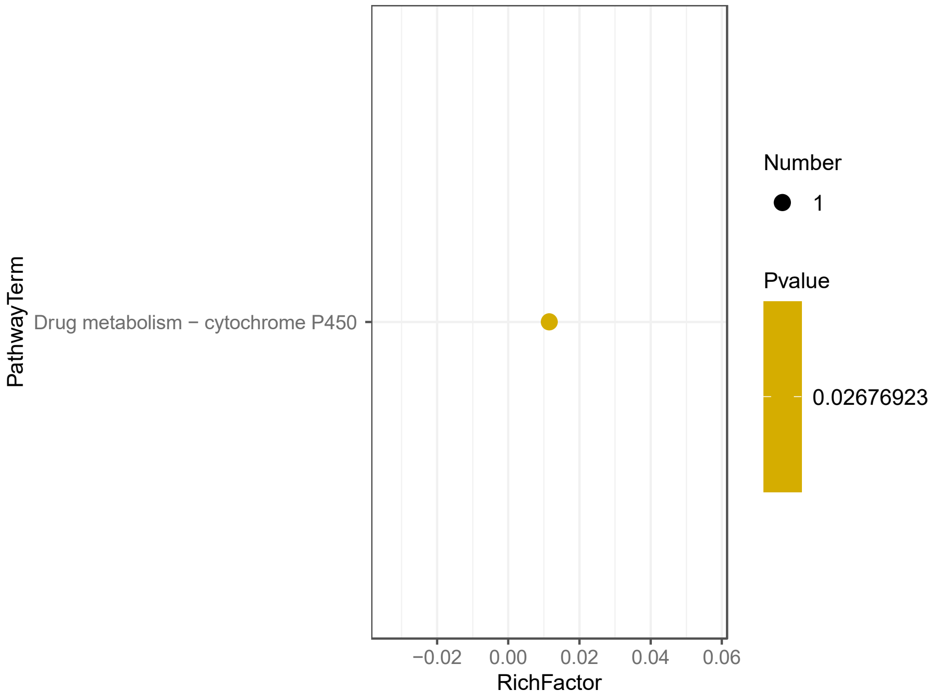
PathwayTerm
Drug metabolism − cytochrome P450
−0.02
0.00
0.02
0.04
0.06
RichFactor
Number
1
Pvalue
0.02676923
